# Inositol polyphosphate multikinase deficiency leads to aberrant induction of synaptotagmin-2 in the forebrain

**DOI:** 10.1186/s13041-019-0480-1

**Published:** 2019-06-20

**Authors:** Jina Park, Seung Ju Park, Seyun Kim

**Affiliations:** 1 Department of Biological Sciences, Korea Advanced Institute of Science and Technology (KAIST), Daejeon, 34141 South Korea; 2 KAIST Institute for the BioCentury, KAIST, Daejeon, 34141 South Korea

**Keywords:** Inositol polyphosphate, IPMK, Transcriptome, Synaptotagmin-2

## Abstract

Inositol polyphosphate multikinase (IPMK), the key enzyme responsible for the synthesis of higher inositol polyphosphates and phosphatidylinositol 3, 4, 5-trisphosphate, is known to mediate various biological events, such as cellular growth and metabolism. Conditional deletion of IPMK in excitatory neurons of the mouse postnatal forebrain results in enhanced extinction of fear memory accompanied by activation of p85 S6 kinase 1 signaling in the amygdala; it also facilitates hippocampal long-term potentiation. However, the molecular changes triggered by IPMK deletion in the brain have not been fully elucidated. In the present study, we investigated gene expression changes in the hippocampal region of IPMK conditional knockout (cKO) mice by performing genome-wide transcriptome analyses. Here we show that expression of synaptotagmin 2 (Syt2), a synaptic vesicle protein essential for Ca^2+^-dependent neurotransmitter release, is robustly upregulated in the forebrain of IPMK^cKO^ mice. Compared to wild-type mice, in which weak Syt2 expression was detected in the forebrain, IPMK^cKO^ mice showed marked increases in both Syt2 mRNA and protein expression in the hippocampus as well as the amygdala. Collectively, our results suggest a physiological role for IPMK in regulating expression of Syt2, providing a potential underlying molecular mechanism to explain IPMK-mediated neural functions.

**Electronic supplementary material:**

The online version of this article (10.1186/s13041-019-0480-1) contains supplementary material, which is available to authorized users.

## Main text

Hydrolysis of phosphatidylinositol 4,5-bisphosphates (PIP_2_) by phospholipases activated in response to cellular stimulation produces inositol 1,4,5-trisphosphate (IP_3_), which mediates release of Ca^2+^ from the endoplasmic reticulum into the cytosol. Studies of the biochemical fate of IP_3_ have unveiled the biosynthetic metabolism of highly phosphorylated IPs [1]. Among the many inositol phosphate kinases, inositol polyphosphate multikinase (IPMK) is the key enzyme responsible for converting IP_4_ into IP_5_ [1, 2]. Thus, IPMK deletion abolishes the formation of IP_5_, IP_6_ and IP_7_, underscoring the essential role of IPMK in generating all highly phosphorylated IP species that have drawn attention as signaling metabolites [1, 2]. In addition to its phosphatidylinositol 3-kinase activity [3], IPMK exerts non-catalytic actions through direct binding to various protein targets, including mechanistic target of rapamycin and transcriptional regulators, such as CREB-binding protein, serum response factor, p53 and steroidogenic factor-1, positioning IPMK as a multifunctional signaling hub in the coordination of cellular growth, apoptosis, and gene expression [4, 5]. In a previous study, our group reported that conditional knockout of *Ipmk* (IPMK^cKO^) in excitatory neurons of the postnatal brain using *CaMKII-Cre* transgenic mice selectively enhances fear extinction accompanied by activation of amygdala p85 S6 kinase signaling and facilitation of hippocampal long-term potentiation [6]. However, whether postnatal deletion of IPMK in excitatory neurons impacts gene expression profiles remains obscure.

**Fig. 1:**
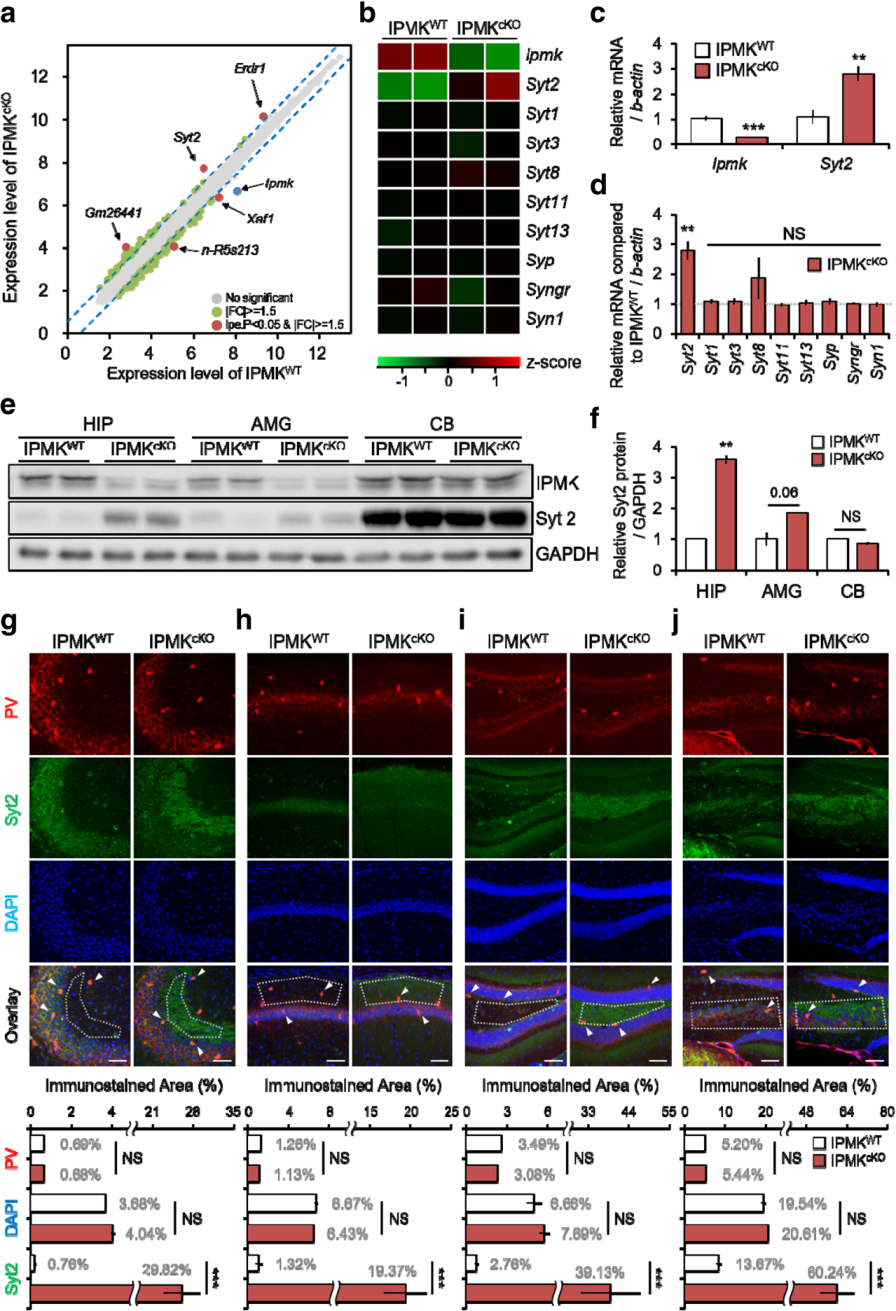
IPMK deletion triggers dynamic changes in synaptotagmin-2 gene and protein expression specifically. **a** Scatter plot shows five genes found to differ in the hippocampus of naive IPMK^cKO^ mice relative to control IPMK^WT^ mice. The x-axis represents differentially expressed genes of IPMK^WT^ mice and the y-axis is that of IPMK^cKO^ mice. The cutoffs for 1.5-fold deviation are indicated by blue lines, respectively. Small gray dots represent sequences with no significant changes, green dots sequences differed genes with no significant (*P* ≥ 0.05). Red dots sequences significantly up- or down-regulated (*P* < 0.05). *n* = 3 (IPMK^WT^) and 3 (IPMK^cKO^) (**b-d**) Levels of major synaptotagmin isoforms and synaptic genes were measured using hippocampal samples obtained from IPMK^WT^ and IPMK^cKO^ mice. **b** Cluster analysis of differentially-expressed genes. The horizontal axis displays individual samples, while the vertical axis displays the expressed genes by their z-scores. Red = increased, green = decreased. **c, d** Quantitative real-time PCR analyses were performed. mRNA expression of the *Ipmk* and *Syt2* were measured (**c**). Levels of synaptotagmin isoforms *Syt1*, *Syt3*, *Syt8*, *Syt11*, *Syt13*, and other synaptic components, *Syp*, *Syngr*, *Syn1* were measured (**d**). In all bar graphs, amounts of mRNA were normalized to those from hippocampus of IPMK^WT^. *n* = 3 (IPMK^WT^) and 4 (IPMK^cKO^) (Student’s *t*-test; NS, *P* ≥ 0.05; **P < 0.01; ***P < 0.001) (**e**) Representative Western blots of IPMK, Syt2, and GAPDH proteins in each mouse hippocampus, amygdala, and cerebellum were presented. **f** All intensities of Western blot bands were quantified using ImageJ software. GAPDH was used as the loading control for quantification. *n* = 3 (IPMK^WT^) and 4 (IPMK^cKO^) (Student’s *t*-test; NS, *P* ≥ 0.05; **P < 0.01) (**g-j**) Immunostaining of hippocampal sections from CA3 (**g**), CA1 (**h**), and DG (**i, j**) of IPMK^WT^ and IPMK^cKO^ mice. Top, representative confocal images were stained by Parvalbumin (red), Syt2 (green), and DAPI (blue). Scale bars, 100 μm. PV positive neurons are indicated by arrowheads. Bottom, Levels of PV, Syt2, and DAPI were quantified. Signals from dashed areas were measured by using using ImageJ software. *n* = 5 (IPMK^WT^) and 5 (IPMK^cKO^) (Student’s *t*-test; NS, *P* ≥ 0.05; ***P < 0.001) In all experiments, IPMK^WT^ littermates served as controls for IPMK^cKO^ mice. HIP, hippocampus; AMG, amygdala; CB, cerebellum. Data are presented as the mean ± SE

To investigate the genome-wide molecular events that occur in the IPMK^cKO^ mouse brain, we analyzed the hippocampal transcriptome of behaviorally naive mice using a microarray technique. This analysis revealed two downregulated genes (*n-R5s213*, *Xaf1*) and three upregulated genes (*Syt2, Erdr1*, *Gm26441*) that were differentially expressed between IPMK^cKO^ mice and littermate controls (Fig. 1a, Additional file 1: Table S1). One of the most strongly upregulated transcripts in the IPMK^cKO^ hippocampus was *Syt2*, which encodes synaptotagmin 2 (Fig. 1a). Our microarray analysis showed no changes in the expression of other Syt isoforms except Syt2 (Fig. 1b). Synaptotagmins are C2 domain-containing Ca^2+^-binding proteins that act as essential players in synaptic vesicle cycling, which is central to synaptic plasticity, learning, and memory [7]. Syt1 is also well known as the major Ca^2+^ sensor for transmitter release at excitatory forebrain synapses [8]. Syt2 exhibits the highest homology with Syt1 and has similar characteristics, allowing it to functionally replace Syt1 [9]. The most notable distinction between Syt1 and Syt2 is their differential expression: the levels of Syt2 are extremely low in the forebrain, where Syt1 is highly expressed, whereas Syt2 is abundantly expressed in the hindbrain and spinal cord [10].

To confirm the results of our microarray analysis, we performed quantitative real-time polymerase chain reaction (PCR) using hippocampal mRNA samples from IPMK^cKO^ and control mice. We found that Syt2 was significantly upregulated in IPMK^cKO^ mice, but observed no changes in other Syt isoforms or synaptic cycling regulators, including Syt1, synapsin, and synaptophysin (Fig. 1c, d). We further found that increases in Syt2 mRNA expression were accompanied by significant elevations in Syt2 protein levels in the hippocampus and amygdala of the IPMK^cKO^ mouse brain (Fig. 1e, f), but not in the cerebellum, in which IPMK was not deleted (Fig. 1e). We further observed that Syt2 levels were abnormally high in the hippocampus and amygdala of IPMK^cKO^ mice that underwent fear conditioning and extinction tasks (Additional file 3: Figure S1).

Because it is known that Syt2 is expressed in GABAergic nerve terminals of parvalbumin (PV) interneurons in the hippocampus and cortex [11, 12], we next examined localization patterns of Syt2 in the forebrain of IPMK^cKO^ mice. Immunohistochemical analyses showed that the Syt2 staining pattern in PV neurons was not altered in the IPMK^cKO^ hippocampus compared with controls (Fig. 1g-j, Additional file 4: Figure S2a), indicating that IPMK deletion does not influence PV neuron populations. This result is consistent with our previous report showing that the balance between excitatory and inhibitory neuronal populations is unchanged by postnatal deletion of IPMK [6]. Importantly, increased expression of Syt2 from the IPMK^cKO^ hippocampus was not detected in PV-positive inhibitory neurons (Fig. 1g-j). We found that elevated Syt2 levels were markedly expanded in broad regions of vGLUT1-positive excitatory neurons within the IPMK^cKO^ hippocampus such as CA3 region (Additional file 4: Figure S2b). Hence, the aberrant upregulation of Syt2 in the IPMK^cKO^ forebrain appears to occur in a cell-autonomous manner within IPMK-deleted excitatory neurons.

In this study, we identified *Syt2* as a gene that is robustly upregulated in IPMK^cKO^ excitatory neurons, suggesting that IPMK is a key player in regulating Syt2 expression in the forebrain. Syt2 acts as a presynaptic Ca^2+^ sensor to drive fast synchronous fusion of synaptic vesicles. With a high sequence homology, Syt1 and Syt2 are structurally and functionally similar, but not identical. Syt2 exhibits its unique kinetic properties in that Syt2 mediates slower vesicle fusion kinetics than Syt1 with a slightly lower affinity for Ca^2+^ than Syt1 [9]. This selective and aberrant induction of Syt2 in the absence of IPMK may lead to functional alterations in synaptic plasticity, thereby establishing a mechanistic basis for IPMK^cKO^ mouse phenotypes, such as enhanced hippocampal long-term potentiation as well as improved fear extinction [6]. Although it has been suggested that DNA methylation [13] and calmodulin signaling [14] mediate the tight suppression of Syt2 expression in the forebrain, our understanding of the molecules that mediate the control of Syt2 expression has been limited. Future studies will elucidate in greater detail how nuclear and signaling actions of IPMK contribute to the transcriptional regulation of Syt2. The recent finding that the IPMK downstream product, 5-IP_7_, inhibits synaptic vesicle exocytosis through direct binding to Syt1 [15] argue for additional investigations of interactions among networks of synaptic vesicle cycling, gene expression, and IP metabolism. Our discovery that IPMK fine-tunes Syt2 expression in the forebrain highlights the importance of fully establishing neural functions of IPMK and offers insights into the treatment and management of psychiatric diseases such as post-traumatic stress disorder.

## Additional files

Additional file 1: **Table S1** List of genes whose pattern of expression was different in the hippocampal tissues of IPMK^cKO^ mice. (DOCX 14 kb)

Additional file 2: Materials and Methods. (DOCX 20 kb)

Additional file 3: **Figure S1** Syt2 was upregulated in the hippocampus and amygdala after fear conditioning and extinction tests. (DOCX 286 kb)

Additional file 4: **Figure S2** Expression patterns of Syt2 in the hippocampus. (DOCX 2499 kb)

## Data Availability

The datasets used and/or analyzed during the current study are available from the corresponding author on reasonable request. Materials and methods are presented in Additional file 2.
