# Supplementary material for: Inositol polyphosphate multikinase deficiency leads to aberrant induction of synaptotagmin-2 in the forebrain
Source: Mol Brain. 2019 Jun 20;12:58. doi: 10.1186/s13041-019-0480-1 (PMC6584979; doi:10.1186/s13041-019-0480-1)
Supplement: Supplementary file 1 — Table S1 List of genes whose pattern of expression was different in the hippocampal tissues of IPMKcKO mice. (DOCX 14 kb) [file 13041_2019_480_MOESM1_ESM.docx]

**Table 1. List of genes whose pattern of expression was different in the hippocampal tissues of IPMK^cKO^ mice.**

| ProbeID | Gene Symbol | Gene Description | Fold change | LPE p value |
| --- | --- | --- | --- | --- |
| 17217536 | Syt2 | synaptotagmin II | 2.425935 | 5.4949 E-08 |
| 17214823 | n-R5s213 | nuclear encoded rRNA 5S 213 | -2.048896 | 0.023748407 |
| 17252341 | Xaf1 | XIAP associated factor 1 | -1.801761 | 0.009795558 |
| 17545407 | Gm26441 | predicted gene, 26441 | 2.378014 | 0.013320497 |
| 17546774 | Erdr1 | erythroid differentiation regulator 1 | 1.824941 | 0.001950217 |
