# Supplementary material for: Inositol polyphosphate multikinase deficiency leads to aberrant induction of synaptotagmin-2 in the forebrain
Source: Mol Brain. 2019 Jun 20;12:58. doi: 10.1186/s13041-019-0480-1 (PMC6584979; doi:10.1186/s13041-019-0480-1)
Supplement: Supplementary file 2 — Materials and Methods. (DOCX 20 kb) [file 13041_2019_480_MOESM2_ESM.docx]

**Materials and Methods**

**Mice**

Forebrain excitatory neuron-specific IPMK knockout mice were generated by crossing floxed Ipmk mice (Ipmk^f/f^) with *CaMKII-Cre* mice, as described previously (Park et al., 2019). Briefly, all IPMK cKO (Ipmk^f/f^;*CaMKII-Cre*) used for experiment with their littermates, IPMK WT (Ipmk^f/f^), which are controls. Animals were bred and housed under specific pathogen-free conditions in a 12 h light-dark schedule, and their food and water were provided ad libitum. Animal protocols were performed in accordance with guidelines approved by the Korea Advanced Institute of Science and Technology Animal Care and Use Committee.

**Microarray analysis**

Microarray experiments were carried out by Macrogen (Korea). Mouse brain hippocampi were dissected immediately after decapitation, and stored at RNAlater Stabilization Solution (Thermo). Four high quality samples containing highest amount of isolated RNA were selected from each experimental group using RNeasy Mini Kit (Qiagen), and stored at – 80 °C until used. For check quality of RNA, RNA purity and integrity were evaluated by ND-1000 Spectrophotometer (NanoDrop), and Agilent 2100 Bioanalyzer (Agilent Technologies). The Affymetrix Whole transcript Expression array process was executed according to the manufacturer's protocol (GeneChip Whole Transcript PLUS reagent Kit). DNA was synthesized using the GeneChip WT (Whole Transcript) Amplification kit as described by the manufacturer. The sense cDNA was then fragmented and biotin labeled with TdT (terminal deoxynucleotidyl transferase) using the GeneChip WT Terminal labeling kit. Approximately 5.5 μg of labeled DNA target was hybridized to the Affymetrix GeneChip Mouse 2.0 ST Array at 45 °C for 16 h. Hybridized arrays were washed and stained on a GeneChip Fluidics Station 450 and scanned on a GCS3000 Scanner (Affymetrix). Signal values were computed using the Affymetrix® GeneChip™ Command Console software. Raw data were deposited in GEO (Gene Expression Omnibus) database under the accession number GSE131748. Raw data were extracted automatically in Affymetrix data extraction protocol using the software provided by Affymetrix GeneChip® Command Console® Software (AGCC). After importing CEL files, the data were summarized and normalized with robust multi-average (RMA) method implemented in Affymetrix® Expression Console™ Software (EC). We exported the result with gene level RMA analysis and performed the differentially expressed gene (DEG) analysis. Statistical significance of the expression data was determined using fold change and LPE test in which the null hypothesis was that no difference exists among groups. False discovery rate (FDR) was controlled by adjusting p value using Benjamini Hochberg algorithm. For a DEG set, Hierarchical cluster analysis was performed using complete linkage and Euclidean distance as a measure of similarity. Gene-Enrichment and Functional Annotation analysis for significant probe list was performed using Gene Ontology (www.geneontology.org/) and KEGG (www.genome.jp/kegg/). All data analysis and visualization of differentially expressed genes was conducted using R 3.1.2 (www.r-project.org).

**Quantitative PCR**

The microarray data were validated utilizing SYBR Green-based real-time quantitative polymerase chain reaction (qPCR) performed. For the qPCR, mouse brain hippocampi were dissected out and stored at RNAlater Stabilization Solution. Later, samples are homogenized in TRI Reagent (Molecular Research Center), and total RNA of hippocampi was extracted according to the manufacturer’s methods. Reverse transcription for 3 μg of each RNA sample was performed with Superscript III (Invitrogen). qPCR analyses were carried out using the SYBR Green Master Mix (Toyobo) and the StepOnePlus Real-Time PCR System (Applied Biosystems). Cycling conditions were 95 °C for 10 min, followed by 40 cycles of 95 °C for 15 s, 58 °C for 30 s, and 72 °C for 30 s. A melting curve analysis was performed to verify the presence of one gene-specific peak and the absence of primer-dimer peaks. 25 Expression levels of genes were normalized to that of beta-actin gene and are presented as fold changes over baseline using the Comparative Ct method (ΔΔCt method). Primers were designed using the Primer-BLAST tool (NCBI) with murine RefSeq database. A list of primers for quantitative PCR are shown below.

| Gene |  | Sequence |
| --- | --- | --- |
| Actb | Forward | 5′-TGGTACCACCATGTACCCAGGCAT-3' |
|  | Reverse | 5′-ACAGAGTACTTGCGCTCAGGAGGAG-3' |
| Ipmk | Forward | 5′-CCAAAATATTATGGCATCTG-3′ |
|  | Reverse | 5′-TATCTTTACATCCATTATAC-3′ |
| Syt2 | Forward | 5′-GGCGGCGAGATGTGATACT-3' |
|  | Reverse | 5′-AGAGGGTGTCTATGATGGCATCAA-3' |
| Syt1 | Forward | 5′-CAAAAGTCCACCGGAAAACC-3' |
|  | Reverse | 5′-TTGCCACCTAATTCCGAGTATG-3' |
| Syt3 | Forward | 5′-CGAACTGCGGATCAGAGGAT-3' |
|  | Reverse | 5′-GGACGATGCCACAGAATGTC-3' |
| Syt8 | Forward | 5′-CCTCCAAGAAAGGCACGACTA-3' |
|  | Reverse | 5′-GCTGGCTAACGGGAACCA-3' |
| Syt11 | Forward | 5′-GACACTTGCCGAAGATGGATATC-3' |
|  | Reverse | 5′-TGCGTTTTCTGCCGTAGTAGA-3' |
| Syt13 | Forward | 5′-CTCCTTAAGTTCCCGGACATCT-3' |
|  | Reverse | 5′-AGGTGTAGTCTGCGTAGTTGATGAC-3' |
| Syp | Forward | 5′-TGACTACTCCTCCTCGGCTG-3' |
|  | Reverse | 5′-CTTCACATCGGACAGGCCTT-3' |
| Syngr | Forward | 5′-TGCTTCCTGACCAACCAGTG-3' |
|  | Reverse | 5′-CAGCCTTGTAGCGCTGGTA-3' |
| Syn1 | Forward | 5′-GAGCAGATTGCCATGTCTGA-3' |
|  | Reverse | 5′-CACTGCGCAGATGTCAAGTC-3' |

**Western blotting**

As described [6], protein lysates of hippocampus, amygdala, and cerebellum were prepared in three group (naive, post-fear conditioning, post-fear extinction) of IPMK^WT^ and IPMK^cKO^ mice. To prepare protein lysates expose to fear conditioning and fear extinction test, mice were euthanized by decapitation 30 min after test and then homogenized in a lysis buffer (1% NP-40, 137 mM NaCl, 20 mM Tris-HCl (pH 8.0), 2 mM EDTA, 10% glycerol, 20 mM NaVO_4_, 10 mM sodium pyrophosphate, 100 mM sodium fluoride, 20 mM PMSF) containing protease inhibitor cocktail (Roche). Generally, 20 μg of protein lysates were separated by size, blotted with primary and secondary antibodies. The HRP signals were visualized with Clarity ECL substrate (Bio-Rad) and measured using ChemiDoc (Bio-Rad). For quantification of immunoblots, ImageJ (NIH) software was used. GAPDH was used as a loading control for quantification.

**Immunohistochemistry**

Immunohistochemistry was performed according to standard techniques, as described previously (Park et al. 2019). Briefly, floating brain sections (30 μm) were obtained using a cryostat and used for staining. Sections were permeabilized and incubated in PBS with 0.3% Triton X-100, then blocked by 5% goat serum in PBS. Incubation with primary antibodies was performed at room temperature overnight (rabbit anti-Syt2, 1:200, Synaptic systems; mouse anti-Parvalbumin, 1:200, Swant; chicken anti-vGLUT1, 1:200, Synaptic systems). Next day, sections were incubated with FITC- or TRITC-conjugated secondary antibodies (1:400 in 0.3% Triton X-100 in PBS). After three times of wash, brain sections were gently mounted onto glass slides. Images were acquired using a confocal laser scanning microscope (LSM780, Zeiss). For quantification of Syt2, PV, and DAPI signals (Fig. 1g-j), confocal microscopic images were analyzed using ImageJ (NIH) program.

**Antibodies**

The commercial primary antibodies were as follows: GAPDH (sc-32233, Santa Cruz Biotechnology), vGLUT1 (135303, Synaptic systems), and Parvalbumin (235, Swant). Two Syt2 antibodies are used -Synaptotagmin 2 (OSS00023W; Thermo Fisher) for Western blotting, Synaptotagmin 2 (105223; Synaptic systems) for immunohistochemistry. The anti-IPMK antibody used was a custom rabbit polyclonal antibody. It raised against a mouse IPMK peptide corresponding to amino acids 295-311 (SKAYSTHTKLYAKKHQS; Covance) containing an added N-terminal cysteine.

**Statistical analysis**

Differences between averages were analyzed using a two-tailed Student’s t test. All data were expressed as means ± SE. Statistical significance was set at *P* < 0.05
