# Supplementary material for: Inositol polyphosphate multikinase deficiency leads to aberrant induction of synaptotagmin-2 in the forebrain
Source: Mol Brain. 2019 Jun 20;12:58. doi: 10.1186/s13041-019-0480-1 (PMC6584979; doi:10.1186/s13041-019-0480-1)
Supplement: Supplementary file 3 — Figure S1 Syt2 was upregulated in the hippocampus and amygdala after fear conditioning and extinction tests. (DOCX 286 kb) [file 13041_2019_480_MOESM3_ESM.docx]

**Figure S1. Syt2 was upregulated in the hippocampus and amygdala after fear conditioning and extinction tests.**

(a-d) Protein lysates of hippocampus (a) and amygdala (c) were prepared 30 min after the fear conditioning or extinction test, and the protein levels were assessed by Western blotting. (b, d) All intensities of Western blot bands were quantified using ImageJ software. GAPDH was used as the loading control for quantification. *n* = 3 (IPMK^WT^) and 4 (IPMK^cKO^) (Student’s *t*-test; **P* < 0.05; ****P* < 0.001)

**
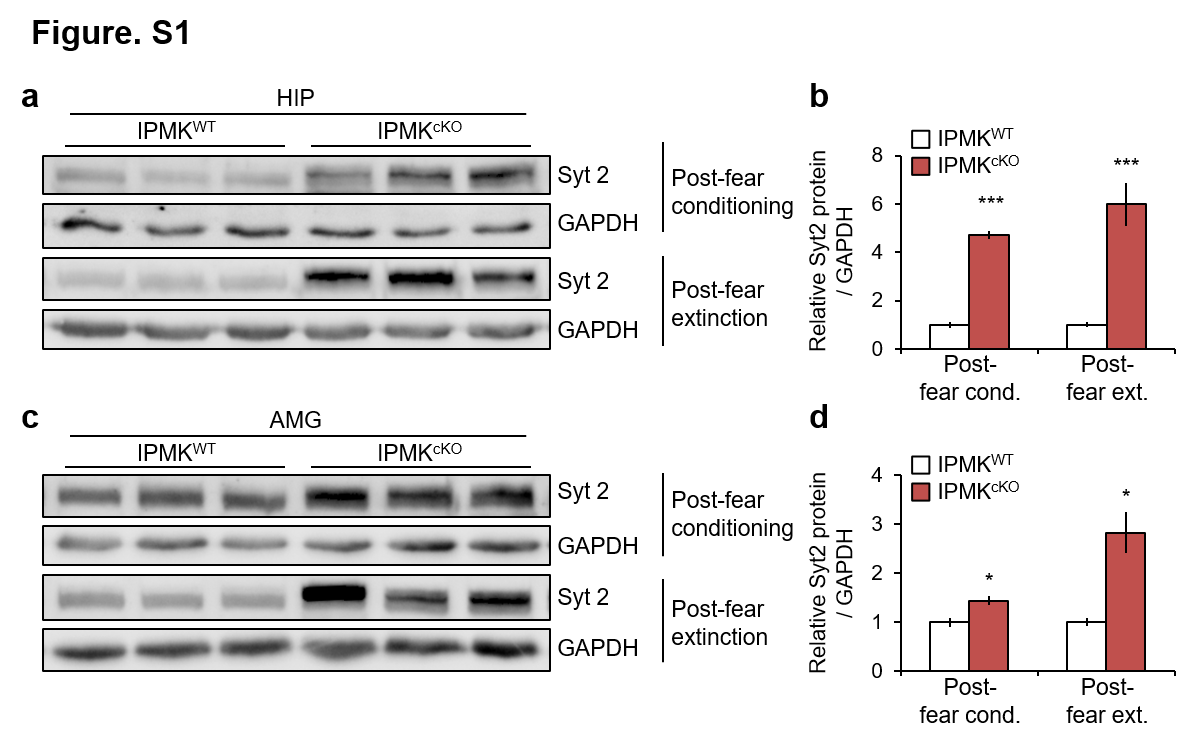
**
