# Supplementary material for: Inositol polyphosphate multikinase deficiency leads to aberrant induction of synaptotagmin-2 in the forebrain
Source: Mol Brain. 2019 Jun 20;12:58. doi: 10.1186/s13041-019-0480-1 (PMC6584979; doi:10.1186/s13041-019-0480-1)

**Figure S2. Expression patterns of Syt2 in the hippocampus.**

(a) Representative confocal images of whole hippocampal sagittal sections of IPMK^WT^ and IPMK^cKO^ mice were immunostained for Parvalbumin (red), Syt2 (green), and DAPI (blue). Scale bars, 250 µm. (b) Immunostaining images from CA3 region of IPMK^WT^ and IPMK^cKO^ mice. Images were stained by vGLUT1 (red), Syt2 (green), and DAPI (blue). Scale bars, 100 µm.


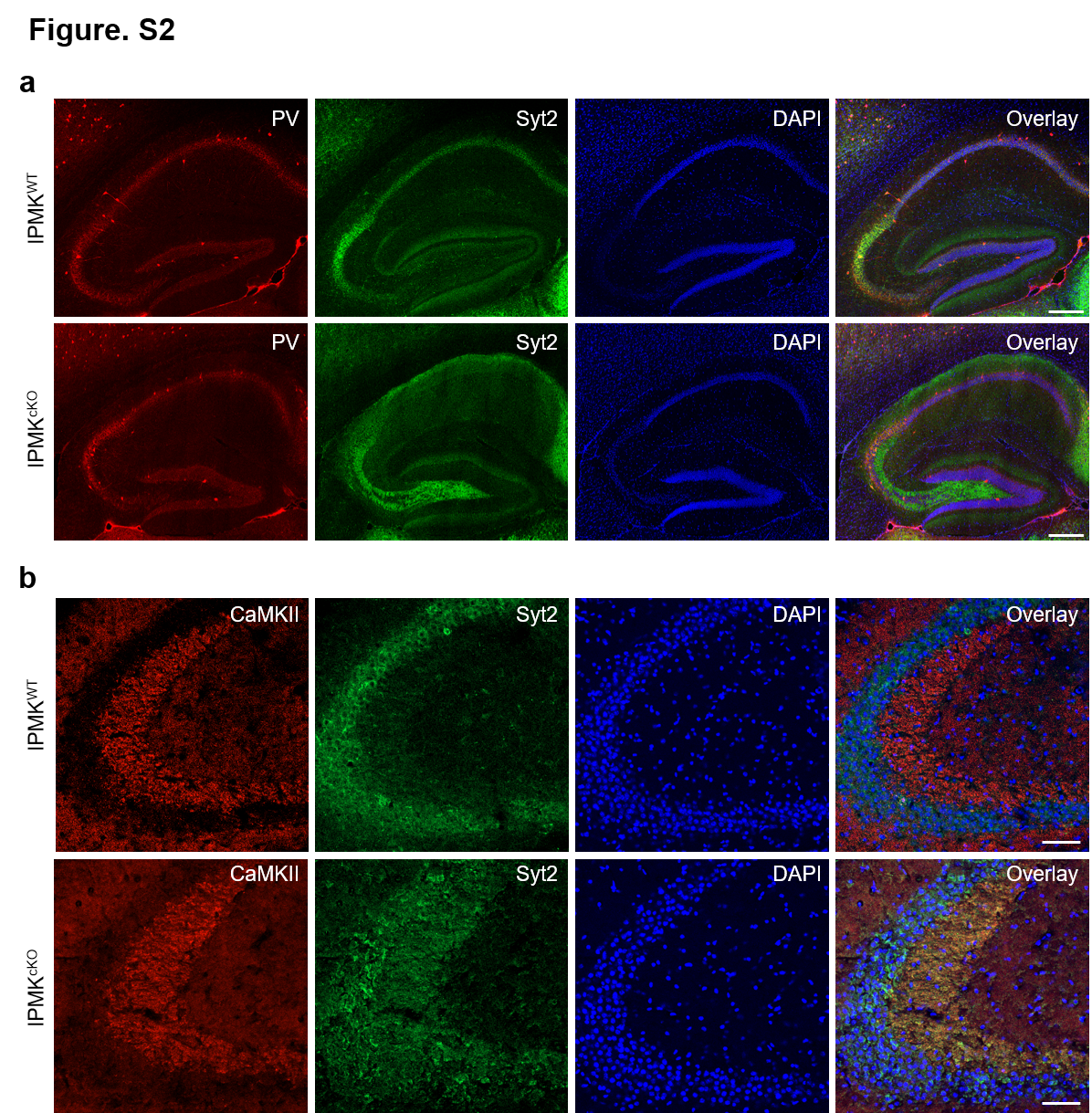

Supplement: Supplementary file 4 — Figure S2 Expression patterns of Syt2 in the hippocampus. (DOCX 2499 kb) [file 13041_2019_480_MOESM4_ESM.docx]
